# Editorial: Redox and Metabolic Circuits in Cancer

**DOI:** 10.3389/fonc.2018.00403

**Published:** 2018-09-26

**Authors:** Salvatore Rizza, Andrea Rasola, Danyelle M. Townsend, Giuseppe Filomeni

**Affiliations:** ^1^Redox Signaling and Oxidative Stress Research Group, Cell Stress and Survival Unit, Center for Autophagy, Recycling and Disease, Danish Cancer Society Research Center, Copenhagen, Denmark; ^2^Department of Biomedical Sciences, University of Padova, Padova, Italy; ^3^Department of Drug Discovery and Pharmaceutical Sciences, Medical University of South Carolina, Charleston, SC, United States; ^4^Department of Biology, University of Rome Tor Vergata, Rome, Italy

**Keywords:** cancer metabolism, redox signaling, tumorigenesis, cancer progression, reactive oxygen species

Biological processes in living cells require a constant supply of energy that primarily derives from the oxidation of biomolecules such as carbohydrates, proteins and lipids. The catabolism of biomolecules relocates electrons to the redox couples NAD^+^/NADH, NADP^+^/NADPH and FAD/FADH2, which represent the principal cofactors of dehydrogenases and reductases used by cells to sustain all endergonic process. While the couple NADP^+^/NADPH is crucial for the antioxidant response and anabolic metabolism, NAD^+^/NADH and FAD/FADH2 convey electrons to the mitochondrial transport chain resulting in the oxygen-dependent production of ATP, the energetic molecule sustaining the activity of the majority of cellular processes. As predictable, there are tight connections between metabolic fluxes, redox balance, oxygen availability, mitochondria function, and turnover.

A peculiar feature of living cells is their extraordinary adaptability to fluctuations in nutrient availability and environmental conditions due to the high plasticity of their biochemical machinery. The back side of the coin emerges, however, in pathologic settings. For instance, cancer cells reprogram the metabolic circuitries in order to sustain their high proliferation rate, invade other tissues, and evade death. An extensive reorganization of cell metabolism is, indeed, a pre-requisite for neoplastic transformation and facilitates tumor progression and metastasis. Cancer cells need to increase the levels of the molecular building blocks for membranes, nucleic acids, and proteins biosynthesis and, at the same time, need to produce elevate levels of ATP to sustain cell proliferation. This metabolic rearrangement, as well as exposure of cancer cells to diverse extracellular environments, inexorably results in the increase of reactive oxygen and nitrogen species (ROS and RNS, respectively), which act as positive modulators of cell growth and are frequently associated with malignant phenotype. The antioxidant capacity of cancer cells, as a consequence, readapts in order to tolerate the increased nitro-oxidative stress, this aspect having profound effects on chemoresistance to drugs. Metabolic rewiring, thus, generates cells that are able to face the adverse conditions they encounter in the process of tumor growth, such as nutrient paucity and nitroxidative stress or anticancer therapies.

The study of the intimate connection between redox and metabolic circuities is becoming a hot field in cancer biology, as it has the potency to provide selective targets for innovative chemotherapeutic tools that interfere with metabolic and/or redox adaptations of cancer cells. In this Research Topic we have assembled a collection of review articles that, we hope, will help the readers obtain a broad overview on different aspects of cancer metabolism and redox signaling. Moreover, we have included a substantial number of original research papers offering new insights on redox/metabolic pathways of cancer cells.

Zulato et al. provide evidence that down-regulation of the liver kinase B1 (LKB1) impacts on cancer cell redox signaling by perturbing the expression of several genes involved in ROS homeostasis. In particular, they found out that LKB1 loss induces NADPH oxidase 1 (NOX1) transcription, thus effecting cell redox state and sustaining tumorigenicity of LKB1-deficient tumors. Indeed, NOX1 inhibition is able to counteract ROS formation, angiogenesis and growth of LKB1-deficient tumor xenografts in mice. Another mechanism by which cancer cells adapt to changes in their redox homeostasis is described in the brief report by Piras et al. They demonstrate how, in aggressive undifferentiated neuroblastoma, the miRNA-494 is involved in the regulation of heme oxygenase 1 (HO-1), a crucial enzyme affecting cell adaptation to oxidative stress and playing an important role in cancer progression and resistance to therapies. In addition, Koundouros and Poulogiannis comprehensively report on the involvement of ROS metabolism and metabolic rewiring in tumorigenesis driven by phosphoinositide the 3-kinase (PI3K)/AKT axis, one of the most frequently deregulated signaling pathways in cancer. The Authors elaborate on different aspects, ranging from the activation of NADPH oxidases (NOXs) to the redox-dependent inactivation of the phosphatase and tensin homolog (PTEN); from the mechanisms through which PI3K/Akt activation helps maintaining redox adaptation of cancer, to the opportunities for therapeutically exploiting redox metabolism in hyperactive PI3K/Akt tumors. The interplay between metabolism rewiring of tumor cells and oncogenic driver mutations is further discussed by De Santis et al., who analyze the crosstalk among mutations in oncogenes (i.e., PI3K/AKT/mTOR, RAS pathway and MYC), tumor suppressors (i.e., p53 and LKB1), cancer cell metabolism and response to therapy.

From a different viewpoint, Stagni et al. focus on the emerging role of a renowned player in the DNA damage response, Ataxia Telangiectasia Kinase (ATM), in redox cancer biology. In this review article, the Authors highlight the complexity of the molecular circuits through which ATM modulates cancer progression by interfering with redox homeostasis and mitophagy in a DNA damage-independent way. ATM mutations, alongside the effects they produce on genome stability, affect mitochondria homeostasis and trigger ROS formation. On the other hand, ATM hyper-activation sustains survival of cancer stem cells by promoting autophagy. Regarding this last process, the role of autophagy in cancer is still debated. It can, indeed, act as a tumor-suppressor during the early stages of tumorigenesis whereas, in established tumors, it sustains the removal of damaged organelles, thus helping cell proliferation, and facilitating drug resistance. The review article from Ichimura and Komatsu discusses on the role of autophagy as major cellular defense mechanism against metabolic and oxidative stress in relation with the Kelch-like ECH-associated protein 1 (Keap1)/nuclear factor (erythroid-derived 2)-like 2 (Nrf2) system, the master regulator of the antioxidant transcriptional response. Autophagy and the Keap1/Nrf2 system are interconnected *via* the phosphorylation of the autophagy receptor protein p62/SQSTM1. The Authors provide an overview on recent findings indicating that p62-Keap1-Nrf2 axis drives cell growth and drug resistance in premalignant cells by promoting metabolic reprogramming.

Besides the established implication of ROS in neoplastic transformation and progression, in the last decades a prominent role for nitric oxide (NO) and *S*-nitrosylation in carcinogenesis has emerged. In this context, Rizza and Filomeni offer a new perspective on the role of denitrosylases, mainly S-nitrosoglutathione reductase (GSNOR), in human cancer, whereas Papaleo's group provides an extensive review article Bignon et al. focusing on the mechanisms of *S*-nitrosylation from a structural and computational point of view, pointing to the main cancer-related targets of *S*-nitrosylation so far identified.

Since the pioneering studies of Otto Warburg, it is clear that most cancer cells preferentially use glycolysis to sustain their high rate of ATP production, even in the presence of normal oxygen tension. Such a metabolic rewiring is commonly referred to as aerobic fermentation, or “Warburg effect,” and it is regulated by several transcription factors, among which the hypoxia-inducible factor 1α (HIF-1α) is one of the most relevant. Dysregulations of HIF-1α expression have been, indeed, implicated in processes such as angiogenesis, energy metabolism, cell survival, and tumor invasion. The oncogenic role of HIF-1α derives from his position at the crossroad between glucose metabolism, oxygen availability, redox stress, and gene transcription regulation. In this research topic, Laitala and Erler elaborate on a new role of HIFs in regulating the extracellular matrix. Cancer progression is, actually, controlled by tumor microenvironment, and hypoxic conditions seem to affect the tumor niche where stromal and cancer cells are in close contact. The original research article by Hlouschek et al. focuses on the resistance of lung cancer cells to radio- and chemotherapy due to chronic cycling hypoxia/reoxygenation stress. They show that this condition is able to trigger the up-regulation of the mitochondrial citrate carrier SLC25A1, impacting on glutathione levels and inducing radio-resistance. Coherently, they provide data suggesting that SLC25A1 inhibitors might sensitize tumor to radiotherapy.

As master regulators of metabolic fluxes, mitochondria play a crucial role in tumor biology. Cannino et al. performed an extensive analysis of the crosstalk of metabolic signals between mitochondria and rest of the cell, focusing on how mitochondrial bioenergetic circuitries can tune the metabolic requirements of cancer cells to the fluctuating environmental conditions. In the short review from Esparza-Moltó and Cuezva, the readers will find an overview on the role of H^+^-ATP synthase and its physiological inhibitor, the ATPase Inhibitory Factor 1 (IF1), whose over-activation in several human cancers is associated with energy metabolism reprogramming and mitochondrial ROS production. Mitochondrial homeostasis has been also investigated by Gibellini et al., who focus on the role of modulation of the mitochondrial Lon protease (LonP) in metastatic colon cancers. They show that LonP1 is poorly expressed in normal mucosa, while it increases gradually from aberrant crypt foci to adenoma, becoming highly abundant in established colorectal cancers. LonP1 expression seems to correlate with mitochondrial dysfunctions, the rate of glycolysis and pentose phosphate pathway, this seemingly enhancing the epithelial-mesenchymal transition.

In this Research Topic, we have also dealt with some therapeutic aspects of cancer cell redox balance and metabolism. Along this line, the original research by Mattarei et al. explores the utility of novel mitochondria-targeted furocoumarin derivatives as possible anti-cancer agents. The Authors synthesized and tested the efficacy of a neo-synthesized coumarin derivative that blocks the potassium channel Kv1.3, this inducing oxidative stress and cytotoxicity in several malignant cells. Lettieri-Barbato and Aquilano elaborate on the effects that diet can have on cancer cells sensitization to conventional cancer therapies, while simultaneously protecting normal cells from their side effects. The Authors review the recent advances in cancer therapy focusing on the effects of adjuvant dietary interventions, and theorize a novel nutritional approach based on moderate ketogenic diets that could be exploited for future pre-clinical research in cancer therapy.

## Author contributions

All authors listed have made a substantial, direct and intellectual contribution to the work, and approved it for publication.

### Conflict of interest statement

The authors declare that the research was conducted in the absence of any commercial or financial relationships that could be construed as a potential conflict of interest.

